# Machine learning: a powerful tool for identifying key microbial agents associated with specific cancer types

**DOI:** 10.7717/peerj.16304

**Published:** 2023-10-23

**Authors:** Jia Feng, Kailan Yang, Xuexue Liu, Min Song, Ping Zhan, Mi Zhang, Jinsong Chen, Jinbo Liu

**Affiliations:** 1 Department of Laboratory Medicine, The Affiliated Hospital of Southwest Medical University, Sichuan Province Engineering Technology Research Center of Molecular Diagnosis of Clinical Diseases, Molecular Diagnosis of Clinical Diseases Key Laboratory of Luzhou, Sichuan, China; 2 Department of Obstetrics, The Affiliated Hospital of Southwest Medical University, Luzhou, Sichuan, China

**Keywords:** Machine learning, Cancer microbiomics, Host-microbe interaction, High-throughput data

## Abstract

Machine learning (ML) includes a broad class of computer programs that improve with experience and shows unique strengths in performing tasks such as clustering, classification and regression. Over the past decade, microbial communities have been implicated in influencing the onset, progression, metastasis, and therapeutic response of multiple cancers. Host-microbe interaction may be a physiological pathway contributing to cancer development. With the accumulation of a large number of high-throughput data, ML has been successfully applied to the study of human cancer microbiomics in an attempt to reveal the complex mechanism behind cancer. In this review, we begin with a brief overview of the data sources included in cancer microbiomics studies. Then, the characteristics of the ML algorithm are briefly introduced. Secondly, the application progress of ML in cancer microbiomics is also reviewed. Finally, we highlight the challenges and future prospects facing ML in cancer microbiomics. On this basis, we conclude that the development of cancer microbiomics can not be achieved without ML, and that ML can be used to develop tumor-targeting microbial therapies, ultimately contributing to personalized and precision medicine.

## Introduction

With the popularization of the concept of “Holobiont” in life and medical science, individual phenotypes are seen as the result of complex interactions resulting from the joint expression of host and related microbial genomes (Simon et al., 2019). In short, almost all functions of macroscopic organisms, including their development, growth, and health, are influenced by the complex microbial communities in which they reside (Foster et al., 2017). It is well known that humans are a host of multiple microbial symbionts, and microbes have co-evolved with human bodies by inhabiting them thereby creating individual habitats that are complex and are specific according to the host physiology (Nallanchakravarthula, Amruta & Ramamurthy, 2021). The efforts of human microbiome projects and advances in diverse omics technologies have revolutionized our understanding of the relationship between humans and microbial symbionts (Parida & Sharma, 2020). Microbiota and host maintain a dynamic equilibrium referred to as eubiosis that actively influences many physiological processes and plays an important role in keeping human health. However, imbalance in the number and types of microbial population leads to ecological imbalance. In this case, the dominant strain can induce chronic inflammation, toxin and carcinogenic metabolites through multiple mechanisms, thereby affecting the host microenvironment and homeostasis (Vimal, Himal & Kannan, 2020). Thus, dysbiosis may directly or indirectly contribute to carcinogenesis in human beings.

The human associated microbiome consists of members from different phylogenetic groups such as bacteria, viruses, fungi, protozoa, archaea and others dominated by bacteria that live symbiotically and nonsymbiotically (Lloyd-Price, Abu-Ali & Huttenhower, 2016). The commensal microbiota colonizes any surface exposed to the surrounding factors, including mucosa and skin (respiratory, gastrointestinal, and urogenital), with the gut being the most densely colonized and widely colonized organ (Loganathan & Priya Doss, 2022). More recent developments show the existence of potentially harboring low-biomass microbial populations in other body sites, initially considered ‘sterile’, such as breast, lung, prostate, bladder, liver, pancreas and blood circulatory systems (Geller et al., 2017; Meng et al., 2018; Nejman et al., 2020; Parhi et al., 2020; Poore et al., 2020; Riquelme, Maitra & McAllister, 2018). Cancer has long been believed to be a complex illness brought on by interactions between the host and its microenvironment (Elinav et al., 2013). Cancer and its associated host microbes are collectively referred to as a Cancer microbiomics (Nallanchakravarthula, Amruta & Ramamurthy, 2021). Numerous studies have demonstrated that each microbial niche may influence cancer promotion through community-level interactions mediated by altered microbiome dysbiosis (Farrell et al., 2012; Sobhani et al., 2011; Xuan et al., 2014), direct interaction of individual members (Koshiol et al., 2016; Pereira-Marques et al., 2019), or *via* secreted or modulated metabolites. Although the causal relationship between microbes and cancer is not absolute, specific microbiome signatures and diversity may be a favorable biomarker for diagnosis and prognosis in patients with cancer.

Increasing evidence suggested that the composition of microbiota changes during the carcinogenesis or development, which gives rise to the promising diagnostic value of microbiome based signatures (Chen et al., 2022; Zhang et al., 2022). Next generation sequencing (NGS) is emerging as a powerful microbiome investigation method, allowing characterization of microbial communities at unprecedented resolution, without prior culturing (Escobar-Zepeda, Vera-Ponce de Leon & Sanchez-Flores, 2015). Due to difficulties identifying biomarkers with standard statistical methods for disease diagnosis, the field has moved to applying predictive ML models for classification of patient phenotypes. ML has great ability to detect informative patterns in the data with limited prior knowledge of the underlying system, which has shown to be useful for identifying key molecular signatures, discovering potential patient stratifications, and particularly for generating models that can accurately predict phenotypes (Li et al., 2022a). Meanwhile, potential biomarkers associated with human disease can be identified through interpretable models (Carrieri et al., 2021; Gou et al., 2021; Wilmanski et al., 2019). Thus, ML has been increasingly applied to cancer microbiomics data to classify samples and predict various outcomes (Fukui et al., 2020; Topcuoglu et al., 2020).

The difficulties in accurate and efficient data analysis have become the immediate challenge that must be tackled for further investigation of the cancer microbiomics. The recently proliferated field of ML sheds new light on such tasks. In the following sections, we will provide an overview of the data sources, methods, potential application, and challenges of ML in cancer microbiomics. This narrative review aims to provide a theoretical basis for promoting the development of cancer microbiomics. At the same time, microbiology researchers and clinicians can quickly understand the current status of microbiology research in cancer and the application of ML ideas.

**Figure 1:**
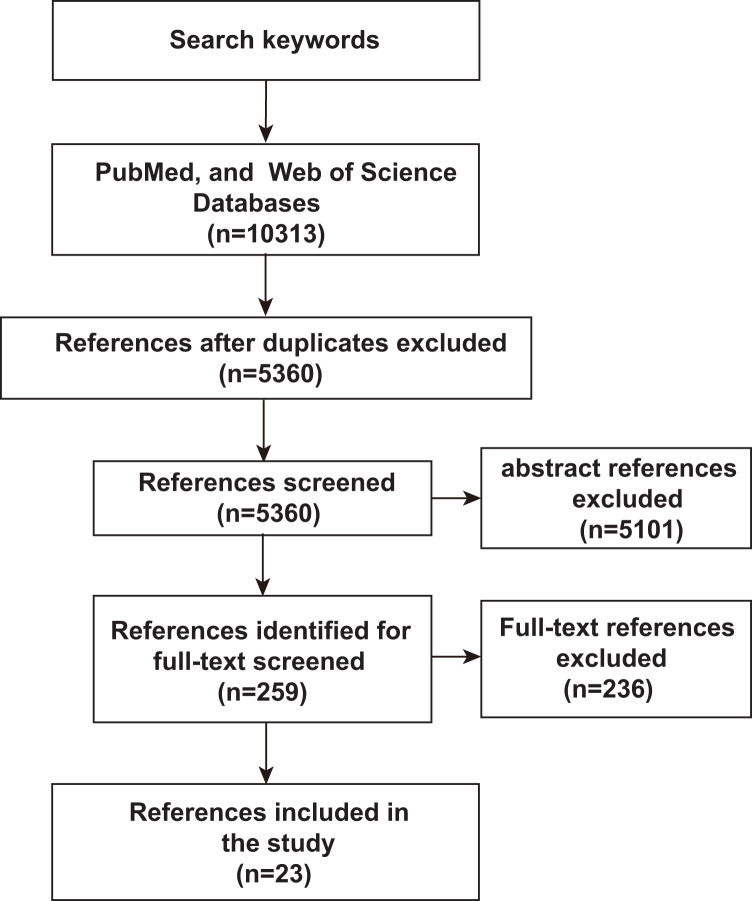
Schematic diagram of literature search. PubMed and Web of Science were searched for keywords, and then after an initial screening of titles and abstracts, the full text was finally scrutinized for inclusion of 23 relevant studies.

To assess an unbiased comprehensive analysis of ML, microbiomics and malignancy correlation studies, we used PubMed and Web of Science to identify relevant literature published before June 2023 to identify and include studies in our analysis. The search terms used were “deep learning or predictive modeling or artificial intelligence or machine learning” AND “tumor or cancer” AND “microflora or microbiome or microbiology or microbial or germ or microorganism or microbe or metagenomics or metagenome”. Only studies published in English or with official English translations were included in this study. The reference lists of eligible studies were manually screened to identify additional relevant literature. As shown in Fig. 1, after an initial screening of titles and abstracts, full-text articles were carefully verified. Included studies met the following criteria: (1) research articles related to microbiomics, (2) the disease studied included malignancy, (3) the study method used ML-related algorithms. Duplicates and studies with unclear ML algorithms were excluded. Twenty-three studies assessing the relevance of microbes, malignancies, and ML were identified by the inclusion and exclusion criteria described above.

## Next generation sequencing technology in microbiome

**Figure 2:**
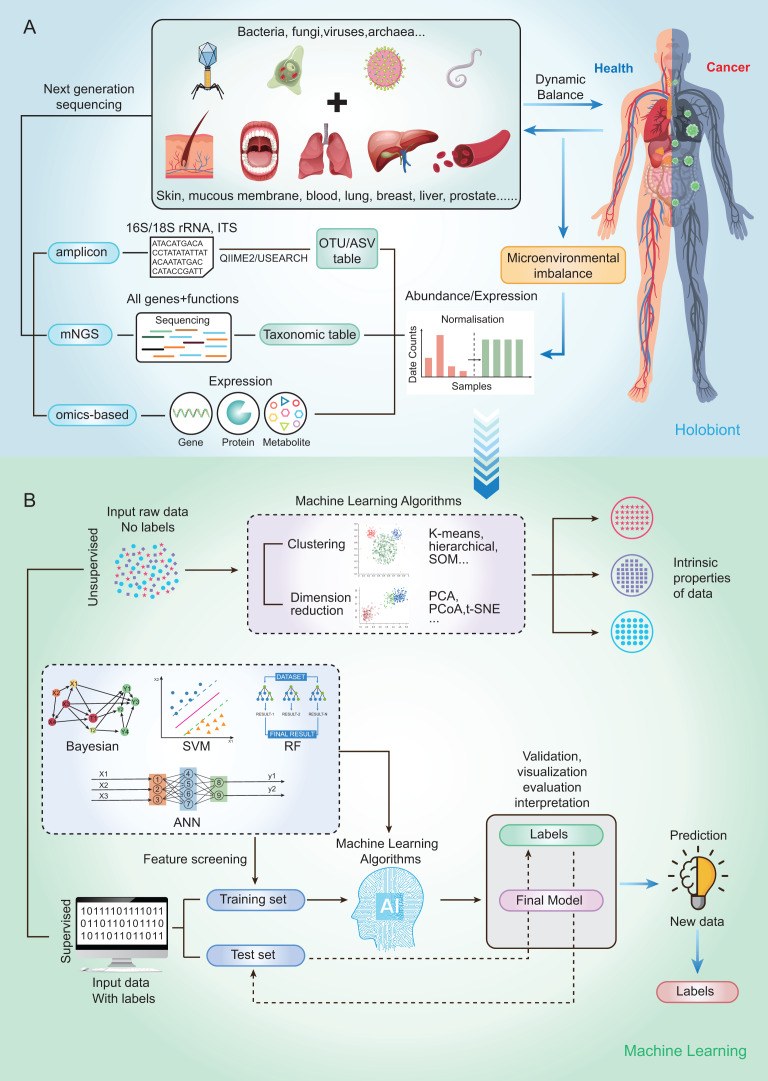
Schematic diagram of disease prediction modeling with microbiome data. (A) The health of the human body is inextricably linked to its microbiota. By subjecting the microbiota of a specific site to next-generation sequencing (amplicon, metagenome, omics-based), it may be possible to make predictions about diseases through the microbiota. (B) Machine learning is usually categorized into supervised (*e.g*., RF, SVM, ANN) and unsupervised (*e.g*., clustering and dimensionality reduction), with very different steps and outputs. The basic steps to build a disease prediction model are: 1. Sequencing data are reasonably divided into training and testing sets, and the data are preprocessed (*e.g*., missing value interpolation, outlier elimination, normalization, or standardization); 2. Parameters are optimized, and appropriate ML algorithms are selected to build the model; and 3. The model needs to be validated, visualized, evaluated, and interpreted.

Currently, microbial community analysis by multiple NGS methods mainly includes amplicon sequencing, metagenomic NGS (mNGS), RNA sequencing and omics-based sequencing (See Fig. 2A). Taxa abundance is the most commonly used feature, as microbiome profiles are assumed to be different in healthy and disease states. Amplicon sequencing is the most common NGS method that could survey almost all bacteria and sample types by targeting the 16s/18s ribosomal RNA (rRNA) housekeeping marker gene to quantify the microbiome composition (Liu et al., 2021). The operational taxonomic units (OTUs) is often used for microbial community analysis, which group sequences into a consensus sequence (the OTU) at a defined sequence similarity threshold by some pipelines (*e.g*., QIIME-uclust, MOTHUR and USEARCH-UPARSE). For the 16S rRNA gene, a threshold of 97% sequence identity is generally used to define OTU to the species level (OTU-97%) (Edgar, 2018), and be widely used in studies. As an alternative to OTUs, amplicon sequencing variants (ASVs) attempt to remodel the exact biological sequences present in the sample using some pipelines (*e.g*., Qiime2-Deblur, DADA2, and USEARCH-UNOISE3) (Callahan, McMurdie & Holmes, 2017). That is, sequences are grouped into the same OTU with a threshold of 100% sequence identity. Compared with OTUs, ASVs has the potential to improve both the sensitivity and specificity of 16S rRNA gene sequence inference. Finally, OTUs/ASVs table is obtained as a helpful and important starting point for most ML prediction approaches to better understand taxonomic variation within microbial communities and its prediction of host traits (Loganathan & Priya Doss, 2022; Zhang, Chen & Wong, 2021). In conclusion, Amplicon sequencing is technically mature enough to obtain sufficient information about microbial community composition in addition to being affordable for large-scale studies (Laudadio et al., 2018). Compared with amplicon sequencing, mNGS could simultaneously examine all genes in all organisms contained in the sample. it can result in species or strain level and has potential to perform metagenomic population functional analysis. However, it is costly and time-consuming. After mNGS, taxonomic table is obtained by comparing to the genome. Then, the taxonomic table can be transferred into input features for ML classifiers and perform prediction for diseases (Liu et al., 2021).

To capture the real-time functional activities of the microbiome, metatranscriptomics, metaproteomics sequencings, and metabolome detection assess the microbial RNA, protein, and metabolite respectively, to extract information on gene/protein/metabolite expression. Thus, gene/protein/metabolite expression is obtained as the input features for downstream analysis and classification by ML.

## Overview of machine learning

In the past 5–6 years, tremendous strides have been made towards using ML for predicting specific phenotypes, from the microbiome data available in public databases (Seneviratne et al., 2020). It is noteworthy that a large volume of high-throughput data has been generated in the microbiome, prompting the association between microbiome and cancer to become increasingly clear, and pushing researchers to use ML-based approaches to predict systematic trends in the cancer microbiomics.

By using a variety of statistical, probabilistic and optimization methods to learn from past experience, ML can build appropriate models from large, unstructured and complex datasets to solve medically relevant problems (Choi et al., 2020). Generally, ML algorithms can be categorized as supervised learning and unsupervised learning. Supervised learning is the most common approach to ML, models in which are optimized (*i.e*., fitted) with a training set of manually labeled input and output data to predict the values of future inputs. Thus, supervised learning algorithms are mainly used to build ML models. For some modeling algorithms (*e.g*., random forest (RF)), the importance of each variable (*e.g*., species and gene) for the model prediction may be estimated for model interpretation or further analysis (Salim et al., 2023). Supervised learning methods are further categorized into regression and classification. Regression returns scores to reflect the possible outcome (a.k.a. class label), while classification directly returns possible outcome the sample can belong to (a.k.a. class label). Among the conventional ML methods, logistic regression (LR), RF (Man et al., 2019), and support vector machine (SVM) (Lyashenko et al., 2020) are the most frequently used supervised learning algorithms. Although ML algorithms such as RFs can handle a large number of features, their accuracy can still be limited in complex datasets (Statnikov et al., 2013). Deep learning (DL) belongs to a subclass of supervised learning, which has attracted increasing attention in microbiome research in recent years due to its unique advantages in processing large amounts of high and complex data. For microbiome data, the input features are relative abundances instead of images, which can be used to build DL models that classify the outcomes into presence or absence of disease state (Xu et al., 2023). The most commonly used form of DL is artificial neural networks (ANN) (Guo et al., 2020).

On the other hand, the unsupervised learning method is essentially used for processing data with no predefined labels. Because no labeled input was provided, the algorithm may explore the intrinsic properties of the data that are not apparent to human. Unsupervised learning optimizes a model by learning the entire dataset and representing it in a more compact way, or by clustering samples together based on the similarity of their features (*i.e*., clustering and dimension reduction) (Raza & Singh, 2021). Dimension reduction methods include t-distributed stochastic neighbor embedding (t-SNE), principal components analysis (PCA) and principal coordinate analysis (PCoA), which have been widely used for omics data visualization and feature extraction by extracting a set of principal variables from high-dimensional feature space (Goodswen et al., 2021). The clustering algorithms, including self-organizing map (SOM), hierarchical clustering and k‑means clustering, are frequently implemented to partition or stratify a set of objects into multiple clusters based on similarities or differences.

The ML methods widely used in microbiome research are well-described by Namkung as published in 2020 (Namkung, 2020). Here, we focus on the implementation of supervised learning methods in human cancer microbiomics studies. So, we briefly sort out the basic steps of building disease prediction models based on microbiome data by analyzing relevant research results in recent years (Fig. 2B). Firstly, the sequencing data were reasonably divided into training and test sets, and the data were preprocessed (such as missing value interpolation, outlier elimination, normalization or standardization). Then the parameters are optimized and the appropriate ML algorithm is selected to build the model. Finally, the model needs to be validated, visualized, evaluated, and interpreted.

## Application of ml for cancer microbiomics

The immense potential of the human microbiome grasped huge attention from the research community of cancer. There has been an explosion in the study of the microbiome in cancer research. In view of the correlation between non-invasive samples and a variety of cancers, as well as the existing microbial multi-omics big data, the current research direction of ML in cancer microbiomics is mainly focused on the development of non-invasive tools for cancer diagnosis, prediction and monitoring. We summarize the main literature on the application of microbial data to build cancer diagnostic models, and the specific research methods and strategies are shown in Table 1. As can be seen from Table 1, a total of 23 studies used ML algorithm to predict cancer phenotypes, among which 12 studies applied RF algorithm, accounting for 52.2%, suggesting that RF algorithm is one of the most widely used ML algorithms in cancer microbiomics.

**Table 1:** The representative applications of machine learning in human cancer microbiomics in recent years.

ML algorithm	Sample type	Data type	Input features	Sample size	External verification	Groups	AUC	Disease	Ref.
GBM	Tissue and blood	Whole genome and transcriptome	Taxonomic table	18,116	169	CA *vs*. BD *vs*. HC	0.891	Pan-cancer	Poore et al. (2020)
RF	Tissue and blood	Whole genome and transcriptome	Taxonomic table	13,664	/	CA *vs*. HC	0.90	Pan-cancer	Xu et al. (2023)
NB	Fecal	16S rRNA	OTUs	90	/	CRC *vs*. CRA *vs*. HC	0.969	CRC	Zackular et al. (2014)
LR	Fecal	Metagenome	Taxonomic table	156	335	CRC *vs*. CRA	0.87	CRC	Zeller et al. (2014)
RF	Fecal	16S rRNA	OTUs	404	/	CRC *vs*. CRA *vs*. HC	0.853	CRC	Baxter et al. (2016)
SVM	Fecal	Metagenome	Taxonomic table	2,424	903	CRC *vs*. HC	0.809	CRC	Pasolli et al. (2016)
NB	Fecal	16S rRNA	OTUs	141	141	CRC *vs*. CRA *vs*. HC	0.994	CRC	Ai et al. (2017)
RF	Oral and fecal	16S rRNA	OTUs	234	/	CRC *vs*. CRA *vs*. HC	0.94	CRC	Flemer et al. (2018)
RF	Fecal	Metagenome	Taxonomic table	156	218	CRC *vs*. HC	0.91	CRC	Koohi-Moghadam et al. (2019)
RF	Fecal	Metagenome	Taxonomic table	60	231	CRC *vs*. HC	1	CRC	Gupta et al. (2019)
LR	Fecal	Metagenome	Taxonomic table	768	203	CRC *vs*. BD	0.92	CRC	Wirbel et al. (2019)
RF	Fecal	Metagenome	Taxonomic table	141	/	CRC *vs*. HC	0.76	CRC	Kishk et al. (2018)
LogitBoost	Fecal	Metagenome	Taxonomic table	696	/	CRC *vs*. BD	0.968	CRC	Bang et al. (2019)
RF	Fecal	16S rRNA	OTUs	242	/	CRC *vs*. HC	0.999	CRC	Qu et al. (2019)
RF	Fecal	16S rRNA	OTUs	46	/	CRC *vs*. HC	0.85	CRC	Trivieri et al. (2020)
LR	Fecal	Metagenome	Metabolites	192	156	CRC *vs*. CRA *vs*. HC	0.98	CRC	Chen et al. (2022)
GBM	Fecal	16S rRNA	OTUs	615	/	CRC *vs*. IBD *vs*. HC	0.80	CRC	Seo et al. (2022)
RF	Lower respiratory tract	Metagenome	Taxonomic table	150	85	CA *vs*. BD *vs*. HC	0.882	Lung cancer	Jin et al. (2019)
SVM	Gut	16S rRNA	OTUs	107	74	CA *vs*. HC	0.976	Lung cancer	Zheng et al. (2020)
RF	Lower respiratory tract	Metagenome	Taxonomic table	60	/	CA *vs*. BD	0.959	Lung cancer	Chen et al. (2023)
LR	Serum	Metagenome	Taxonomic table	350	/	CA *vs*. HC	0.99	Brain tumor	Yang et al. (2020)
RF	Peritoneum	16S rRNA	OTUs	30	/	CA *vs*. BD	0.94	Ovarian cancer	Miao et al. (2020)
RF	Gastric juice	16S rRNA	OTUs	139	/	CA *vs*. HC	1	Gastric cancer	Wei et al. (2023)

**Note:**

CA, cancer; BD, benign diseases; HC, health controls; AUC, area under the curve; operational taxonomic units; CRC, colorectal cancer; CAR, colorectal adenoma; IBD, inflammatory bowel disease; ML, machine learning; RF, random forest; NB, Naïve Bayesian; LR, logistic regression; SVM, support vector machine; GBM, gradient boosting machine; Ref., reference.

### ML algorithms in pan-cancer

Based on the extensive association between microbiome and cancer, and to characterize the cancer-associated microbiome, Poore et al. (2020) re-examined microbial reads from 18,116 samples and 33 cancer types from The Cancer Genome Atlas (TCGA) compendium of whole genome sequencing (WGS; *n* = 4,831) and whole transcriptome sequencing (RNA-Seq; *n* = 13,285) studies. They built the stochastic gradient boosting ML model that was able to distinguish well between and within cancer types and stages. They then performed deep metagenomic sequencing of plasma samples from cancer individuals with prostate, lung, and skin cancer (*n* = 100) and non-cancer, HIV-, healthy controls (*n* = 69). The ML model also could distinguish well healthy from cancer and cancer from cancer in the cell-free microbial profiles. This finding has already shown promise for a universal strategy for cancer diagnosis based on the microbiome.

Coincidentally, Xu et al. (2023) applied a large amount of microbial data from 21 cancer types, including 11,819 tissue samples (metagenomic data and 16S rRNA sequencing data) and 1,845 blood samples (metagenomic data), to construct DeepMicroCancer. The DeepMicroCancer is a set of tissue/blood microbiome based RF and tissue-blood microbiome based transfer learning models that can be used to diagnose a wide range of cancer types. Thereinto, the tissue RF model demonstrated superior predictive performance, with area under the curve (AUC) >0.9 for the prediction of all other cancers except for lymphoid neoplasm diffuse large B-cell lymphoma (only seven samples). The top contributing bacteria *Ralstonia, Tetrasphaera, Nitrospira*, and *Luteimonas* of this RF model were generally distributed in natural environments like soil and water habitats or plant surfaces, while some of which such as *Ralstonia* were proved to cause infection when transferred to hospital settings (Ryan & Pembroke, 2018). In a study by Higuchi et al. (2021), it was also shown that some bacteria such as *Ralstonia* constitute a mesothelioma-specific microbiota that promotes the process of cancer progression. Although the blood RF model has decent performance, which is unsurprisingly not comparable with that of the tissue RF model. The top contributor in the blood RF model is *Herbaspirillum*, which has been reported to be isolated from multiple cancer patients (Chemaly et al., 2015; Gungor et al., 2020; Suwantarat et al., 2015). In addition, the tissue–blood transfer learning (TL) model has a precision of 0.5 or higher for most cancers, and the multiclassification averaged AUC reached 0.89, which performed better than the RF model in predicting cancer types in small samples (Xu et al., 2023). These findings suggest a new class of microbial-based cancer diagnostics, providing a unique opportunity to develop cancer diagnostics.

### ML algorithms in colorectal cancer (CRC)

As routine screening programs for CRC, non-invasive methods such as fecal occult blood testing (FOBT) and carcinoembryonic antigen (CEA) testing, and invasive methods such as high-quality colonoscopy can reduce the risk of death from CRC to some extent (Medical Advisory Secretariat, 2009; Forones & Tanaka, 1999; Waldmann et al., 2021). However, the large-scale use of these methods is limited by the low accuracy of non-invasive methods and the damage caused by invasive methods. The researchers therefore focused on the gut microbiome, a novel non-invasive method of CRC detection, in an attempt to find new potential biomarkers. There is no doubt that CRC is one of the most widely applied cancer types for ML algorithms. This has been summarized in detail in a review published by Zhang, Chen & Wong (2021), and we have found some new investigations regarding CRC diagnosis based on that review as well (see Table 1). It is worth mentioning that almost all CRC related literature in Table 1 used fecal microbiota to establish diagnostic models. With the exception of Kishk et al. (2018) almost all ML models have AUC values of 0.8 and above. This not only reflects the advantages of non-invasiveness and accessibility of stool types, but also reflects the huge representation of CRC characteristics of stool samples. In the following, we will supplement some recent new research in detail.

Due to the abundance of microbial species and quantity, it is particularly important to select suitable dimensionality reduction methods for feature selection prior to modeling. Based on two public data sets, Qu et al. (2019) used single method (correlation-based feature selection and maximum relevance-maximum distance) and multiple dimension-reduction methods (single method combination) to select features. Finally, multiple ML algorithm are utilized for modeling and diagnostic performance evaluation. The results show that the model with RF combined with multiple dimension reduction method has the best performance (AUC = 0.999). In additional, Koohi-Moghadam introduced a novel concept—MetaMarker (https://bitbucket.org/mkoohim/metamarker under GPLv3 license) (Koohi-Moghadam et al., 2019). Unlike the traditional OTU tables, it is effective in identifying unknown bacterial sequences that may be important for disease. Based on the whole-metagenome sequencing of fecal samples, they compared the performance evaluation of RF modeling in multiple national datasets with metaMarker or linear discriminant analysis of effect sizes (LEfSe) markers (Segata et al., 2011). The results showed that MetaMarker has better performance than LEFSe in distinguishing CRC samples from healthy individuals in a multiracial population. Upon pooling biomarkers from MetaMarker and LEfSe, the ML model improved classification performance beyond that of either model and the AUC could reach 0.91.

The colorectal adenoma (CRA) and inflammatory bowel disease (IBD) are the two high-risk diseases for CRC at early screening. CRA is a precancerous lesion, and early diagnosis can effectively prevent the development of CRC (CRA to CRC) (Bray et al., 2017). Furthermore, the increase of some specific harmful bacteria, such as enterotoxigenic *Bacteroides fragilis* and *Escherichia coli*, is associated with the chronic tissue inflammation, release of pro-inflammatory mediators and oncogenic mediators, which may increase the chance of CRC in patients with IBD (inflammation-anisoplasia-cancer) (Quaglio et al., 2022). Zackular et al. (2014) first used the Kruskal-Wallis test to identify OTUs with significant differences, and then performed linear discriminant analysis (LDA) to determine the effect sizes of these specific attributes. The largest differences between adenoma and healthy groups included age, race and five OTUs (including *Clostridiales*, *Clostridium*, *Lachnospiraceae* and *Bacteroides*). The authors used the above screened feature variables to build a Naïve Bayesian (NB) model. It was found that modeling with the NB algorithm could increase the probability of adenoma detection by more than 50 times, with an AUC as high as 0.969. The model showed that when at least one of these five OTUs was not detected, it was a signal for the presence of adenoma. The differential microorganisms identified by their studies are also supported by previous evidence (Castellarin et al., 2012; Kostic et al., 2013; Rubinstein et al., 2013). For example, *Bacteroides fragilis*, a pathogenic variant that is a common commensal, has been shown to directly influence the development of CRC in a mouse genetic model by producing a metalloproteinase toxin (Sears et al., 2008). Similarly, Zeller et al. (2014) developed a CRC diagnostic model using metagenomic data from feces of CRC patients *vs*. CRA patients (Zeller et al., 2014). First, the authors assessed the predictive ability of microorganisms that differed significantly between the two groups and found that individual species already had some predictive ability (AUC up to 0.75). Next, the authors selected the top 22 species to build the last absolute shrinkage and selection operator (LASSO)-LR model, whose AUC could be raised to 0.84. Among those significantly enriched in CRC were *Fusobacterium*, *Porphyromonas asaccharolytica* and *Peptostreptococcus stomatis*. Although it is not clear which microorganisms are directly associated with CRC, recent evidence has shown that Fusobacterium species are prevalent CRC-associated (Castellarin et al., 2012) and tumor-accelerating (Kostic et al., 2012) microorganisms. Meanwhile, when the fecal metagenome was combined with FOBT, the accuracy of the model could be improved (AUC of 0.87). The patients included in the control group of this study were patients with small colorectal adenomas (diameter <10 mm), and thus the model developed in this study is a good predictor of microadenomas that are not easily detected by colonoscopy. In other words, for smaller colorectal adenomas, microbiomics can be used as a complementary test to colonoscopy to reduce the probability of their being missed. In addition, Seo et al. (2022). developed a gradient boosting machine (GBM) model for CRC and IBD differential diagnosis. Model evaluation showed that the IBD risk model (AUC of 0.84, specificity of 74%, and sensitivity of 91%) performed slightly better than the CRC risk model (AUC of 0.80, specificity of 84%, and sensitivity of 71%) for disease classification. In the IBD risk model, the most important genera were *Dorea*, *Blautia*, *Enhydrobacter*. Meanwhile, in the CRC risk model, the most important genera were *Parvimonas*, *Peptostreptococcus*, *Psychrilyobacter*.

Some oral-associated microorganisms have been reported to be present in the fecal and colonic mucosal microbiota associated with CRC (Baxter et al., 2016; Flemer et al., 2017; Zeller et al., 2014), which may also be candidates for CRC biomarkers. Flemer et al. (2018) analyzed microbiota in oral, colonic mucosa, and feces from CRC patients (*n* = 99), colorectal polyp patients (*n* = 32), and healthy controls (*n* = 103). The authors first filtered out features that appeared in less than 5% of the individuals. Then in each iteration of the 10-fold cross-validation, LASSO algorithm was used to select features for 90% of the data set. Finally, The highest accuracy, with an AUC of 0.94, specificity of 95%, and sensitivity of 76%, was achieved when combining fecal and oral microbiota data to create a diagnostic model for RF. This study shows that simultaneous analysis of oral and fecal microbiomes can enhance the diagnostic efficacy of CRC.

CRC carrying the *BRAF*^V600E^ mutation has been reported to have a low response to conventional therapy and a poor prognosis (De Sousa et al., 2013; Ursem, Atreya & Van Loon, 2018). Trivieri et al. (2020) found that unique microbiota signatures can distinguish cases of *BRAF* mutations in CRC. They first studied fecal microbiota characteristics in CRC-carrying mice, a cohort of human CRC subjects, and a tumor-free control group by performing bacterial 16S rRNA sequencing. All of these data confirmed that a distinctive microbiota’s fingerprint could be distinguished between serrated *BRAF*^V600E^ and *BRAF* wt CRC’s patients, with the former strongly resembling healthy subjects. They then detected 10 candidate species that were differentially expressed in the two CRC groups with good predictive potential to distinguish patients with *BRAF*^V600E^ from those with *BRAF* wt. Finally, the authors constructed an RF model based on these bacterial markers, which performed well in discriminating serrated CRC driven by *BRAF* mutation from *BRAF* wild-type CRC cases (AUROC = 0.85, 95% confidence interval [0.69–1.01]), with *Prevotella enoeca* and *Ruthenibacterium lactatiformans* contributing the most. In conclusion, this RF model can differentiate the *BRAF* status of CRC patients, and thus microbiomics may be a potential non-invasive test for it.

However, focusing on one disease may not detect biomarkers specific to that disease because the host’s gut microbial community may be susceptible to a variety of diseases simultaneously (Kinross, Darzi & Nicholson, 2011). Therefore, using the gut microbiota to explore multiple diseases simultaneously may provide a more comprehensive diagnostic value for clinical purposes. Bang et al. (2019) developed diagnostic models for CRC and other diseases (multiple sclerosis, idiopathic arthritis, myalgic myelitis, acquired immunodeficiency syndrome and stroke) from five classification levels (phylum, class, order, family and genus), four classifiers (LogitBoost, SVM, K nearest neighbor and logistic model tree) and two feature selection methods (forward selection and backward elimination). The LogitBoost model performed best when built using genus level and backward elimination, where the diagnostic accuracy for CRC was 96.84%. However, the subset of features selected in this study may not contain all microorganisms associated with the six diseases, which could introduce errors into the study results. The characteristics selected for the study can distinguish these six diseases simultaneously, and PSBM3 is one of the important biomarkers. PSBM3 belongs to a family of bacteria known as *Erysipelotrichaceae* associated with the immune system, and its abundance is positively correlated with tumor necrosis factor alpha levels (Dinh et al., 2015; Kaakoush, 2015; Palm et al., 2014). Not coincidentally, Wirbel et al. (2019) developed a cross-regional CRC diagnostic model by analyzing eight geographically and technologically distinct CRC fecal metagenomic studies. At first, the authors identified 94 differentially abundant microorganisms from the 849 species. Then, among these markers, the analysis was focused on the most important core group consisting of 29 species such as *Fusobacterium*, *Porphyromonas*, *Parvimonas*, *Peptostreptococcus*, *Gemella*, *Prevotella*, and *Solobacterium*. Most of these species are from the *orphyromonas* and *Dialister* genera and the *Clostridiales* order, which is strongly enriched in the CRC group and usually undetectable in controls (including type two diabetes, Parkinson’s disease, and inflammatory bowel disease). Next, the authors applied differential microbial species to the LASSO-LR classifier for CRC diagnostic modeling, which can reach an AUC of 0.92. The results show that this polymicrobial CRC classifier can overcome technical and geographic research differences and has the potential to expand its applicability through validation in other regions.

Metabolic changes can be more directly observed in the state of caner cells than genomic and proteomic changes, and therefore can be used as a promising cancer marker (Patel & Ahmed, 2015). Chen et al. (2022) developed a panel of gut microbiome-associated serum metabolites (GMSM) that can accurately predict CRC and colorectal adenoma from normal healthy population (N) through a combined analysis of serum metabolomic and faeces metagenomic. In this study, they first performed a PCA of differential metabolites (CRC *vs*. CRA, CRA *vs*. N, CRC *vs*. N) in the discovery dataset and found that the pattern was similar in both CRA and CRC patients, while the healthy population could be clearly distinguished from both populations. Subsequently, through an integrated microbiome–metabolome associated analysis, they found that these correlated species-metabolite pairs included bacterial species that were reported to be associated with CRC initiation and progression (such as CRC-promoting *P. micra*, *F. nucleatum*, *Odoribacter splanchnicus* and *Alistipes finegoldii*) and probiotics (such as *Parabacteroides distasonis* and *B. longum*). Based on these differential GMSM, 32 metabolite characteristics were initially screened after 200 LASSO experiments. Of these, eight metabolites were reliably identified in both non-targeted and targeted metabolomics assays, and both assays could accurately distinguish between normal and abnormal colorectal patients in the discovery cohort, with an AUC of 0.95 (95% CI [0.85–1.00]). PCA analysis based on the abundance of eight GMSM was still able to clearly separate normal individuals from abnormal colorectal patients. Then, a GMSM panel-based logistic regression model to predict CRC and CRA was built and yielded an AUC of 0.98 (95% CI [0.94–1.00]) in the modelling cohort. In the validation cohort, the GMSM model was significantly better than the clinical marker CEA (AUC = 0.92 *vs*. 0.72, sensitivity = 83.5% *vs*. 35.8%, specificity = 84.9% *vs*. 86.4%), and in CRA (AUC = 0.84 sensitivity = 63.2%) and early CRC (AUC = 0.93 sensitive = 88.2%) also performed well. In addition, the GMSM model was superior to the FOBT/FIT test (65.2% sensitivity) in detecting CRC. This study confirms the potential application of GMSM in CRC and CRA.

### ML algorithms in lung cancer (LC)

Although the lung was previously thought to be sterile, the presence of a diverse microbiome in the lower respiratory tract has been confirmed (Wang et al., 2021). Based on metagenomic data, Jin et al. (2019) and Chen et al. (2023) analyzed lower respiratory tract microbiome profiles of LC patients, non-malignant lung disease patients, or healthy individuals. They all found that microbiota abundance was significantly lower in the lower respiratory tract of LC patients compared to controls. Furthermore, Jin et al. (2019) developed a RF model based on age, years of smoking, and 11 bacterial species (including *Streptococcus sp. I-P16*, *Prevotella melaninogenica*, *Acidovorax sp. KKS102*, *Corynebacterium urealyticum*, *Streptococcus sanguinis*, *Pseudomonas aeruginosa*, *Streptococcus pseudopneumoniae*, *H. influenzae*, *Campylobacter concisus*, *Bacteroides salanitronis*, and *B. japonicum*) with an AUC of 0. 882. Particularly, *Bradyrhizobium japonicum* was only present in patients with LC, yet *Acidovorax* was only present in patients with lung disease. *Bradyrhizobium japonicum* has been associated with inflammatory bowel disease in previous studies (Bhatt et al., 2013), further suggesting the relevance of inflammation to cancer. Then, Chen et al. (2023) based on five key genera (*Prevotella*, *Klebsiella*, *Pedobacter*, *Mycobacterium*, and *Xanthomonas*) and one tumor marker (neuron-specific enolase) as variables, the best diagnostic model for LC was constructed using the RF classifier (AUC = 0.959). These model-related genera are speculated to be related to LC. For example, *Klebsiella* is usually associated with lung infections (Paudel et al., 2020). *Mycobacterium* is a well-defined pathogen that causes tuberculosis, and a history of tuberculosis has been clinically shown to be strongly associated with an increased risk of LC (Hadifar et al., 2021). *Prevotella* was significantly more abundant in LC patients than in patients with benign lung disease, which is consistent with previous studies (Tsay et al., 2018). *Xanthomonas* has also been reported to have antitumor effects (Ma et al., 2020). These key microorganisms are of great value in the study of cancer mechanisms and have the potential to become new targets for LC therapy.

In addition, the gut microbiota can also be used to develop a diagnostic model for lung cancer. Based on 16S rRNA sequencing data from early-stage LC patients and healthy individuals, Zheng et al. (2020) screened 13 OTUs for SVM modeling using minimum-redundancy maximum-relevancy (mRMR), which had a high diagnostic accuracy for LC (AUC = 0.976) (Alshamlan, Badr & Alohali, 2015; Zheng et al., 2020). Among them, *Roseburia spp*. has been shown to influence immune maintenance and anti-inflammatory effects through multiple metabolic pathways (Louis & Flint, 2009), *Ruminococcus bromii*-related microorganisms promote intestinal health by degrading starch and short-chain fatty acids (Abell et al., 2008), *Streptococcus infantis* modulates lung intrinsic immunity by detoxifying polycycles (Hosgood et al., 2014), and *Veillonella* is associated with Th17-mediated immunity in the lung (Mur et al., 2018). However, this study is only a preliminary study and the detailed link between the enteropulmonary axis and lung immunity or cancer development remains to be further elucidated.

### ML algorithms in other cancers

In previous studies, brain cancer was a disease that could not be linked to the microbiome because microorganisms usually cannot cross the blood-brain barrier, which was present throughout various regions of the brain. However, some nanoscale extracellular vesicles released by microorganisms can cross the blood-brain barrier into the brain (Cattaneo et al., 2017), and thus extracellular vesicle microbiome data in the blood may be a powerful biomarker for assessing brain disease. Yang collected serum specimens from 152 brain cancer patients and 198 healthy controls, and performed genomic analysis of microbial extracellular vesicle components (Yang et al., 2020). This study used the relative abundance of OTUs at the genus level as a model variable. The highest diagnostic performance was achieved when GBM was combined with Logistic regression to build a model with an AUC of 0.99. It was found that *Dialister* and *E. rectale* were significantly decreased in the blood of brain cancer patients, while *Lachnospiraceae NK4A136* was significantly increased. This is consistent with the findings of several previous studies, which suggested that the abundance of *Dialister*, *E. rectale*, and *Lachnospiraceae NK4A136* in the intestine was associated with several neurological disorders (Cattaneo et al., 2017; Jiang et al., 2015; Strati et al., 2017; Vogt et al., 2017). For example, *E. rectale* abundance is lower in amyloid-positive (or negative) patients with mild cognitive impairment (Cattaneo et al., 2017), yet *Dialister* abundance is lower in patients with Alzheimer’s disease, autism, and depression, compared to healthy controls (Jiang et al., 2015; Strati et al., 2017; Vogt et al., 2017). This study is the first to analyze extracellular vesicles in blood as a biomarker for brain cancer diagnosis, and its findings are relevant as a new and accurate method for brain cancer diagnosis.

Due to the absence of effective screening methods for early detection of ovarian cancer at this stage and the fact that early ovarian cancer symptoms are usually reported as non-specific (stomach upset, bloating and constipation), this results in more than 60% of patients being diagnosed at an advanced stage (Gaona-Luviano, Medina-Gaona & Magaña-Pérez, 2020). Miao subjected peritoneal fluid from patients with ovarian cancer and benign ovarian masses to next-generation sequencing assays, and the obtained OTUs were evaluated by a series of feature selection methods, including Lasso coefficients, RF variable significance, distance correlation, t-test, and Mann-Whitney test (Miao et al., 2020). when using the top 18 OTUs in combination with age, BMI and serum tumor markers (cancer antigen 125 and human epididymis protein 4) to build the RF diagnostic model can greatly improve the accuracy of ovarian cancer diagnosis (AUC of 0.94). Through the above ML algorithm analysis, three different microbial characteristics were identified and may be related to the diagnosis and pathogenesis of ovarian cancer. They are anti-inflammatory properties (*Akkermansia* and *muciniphila*), estrogenic response (*Rikenellaceae*) and vascular permeability (*Alphaproteobacteria*). Reaserch has comfirmed that *Akkermansia* has a clear anti-inflammatory effect and may increase immunotherapy effectiveness in cancer patients (van Passel et al., 2011). Rikenellaceae is considered to be fecal microbiota related to ESR1 function, mainly involved in breast cancer prediction, prognosis and metastasis (Carausu et al., 2019; Javurek et al., 2016; Li et al., 2022b).

It has been reported that the development of gastric cancer is associated with microorganisms, and in addition to *H. pylori*, which is currently known to be a high risk factor for gastric cancer, other microorganisms may also serve as potential biomarkers for gastric cancer (Eun et al., 2014; Lertpiriyapong et al., 2014; Sha et al., 2020). Wei et al. (2023) collected gastric juice from healthy individuals (*n* = 61) and gastric cancer patients (early stage *n* = 48, late stage *n* = 30) and sequenced the 16S rRNA V1–V4 region. They adopted six ML algorithms, SVM, RF, LR, Catboost, neural network, and gradient boosting tree, to construct a risk prediction model for gastric cancer, among which the RF prediction model had the highest accuracy of 82.73%. In this model, the bacteria with the highest predictive value were *Streptococcus*, *Lactobacillus* and *Ochrobactrum*. Among them, *Streptococcus* is the bacteria with the highest proportion in early and advanced gastric cancer, which is consistent with the results of Zhou et al. (2022). Study has confirmed that *Streptococcus* can promote the development of gastric cancer in a number of ways, including increasing N-nitroso compounds that cause DNA damage, and regulating the expression of key molecules important in cancer development (San-Millán & Brooks, 2017). In contrast, *Lactobacillus* induces anti-cancer effects by enhancing cancer cell apoptosis and protecting against oxidative stress (Badgeley et al., 2021).

## The challenges and future outlook of ml in cancer microbiomics applications

Although ML has made great achievements in microbiological research, its application in cancer microbiomics still faces many difficulties. Only through in-depth research and analysis of these issues can we facilitate the application of ML in cancer microbiomics and accelerate the pace of its clinical translation. These difficulties are mainly manifested in the following aspects. The first aspect is the amount of data. The accuracy of a ML model is highly dependent on the amount of data it is trained on. Due to some objective factors, the sample size included in modeling is very small, such as expensive genomic sequencing data.. Therefore, in microbiome research, the current situation of reducing sequencing costs and increasing the amount of sequencing data is still worthy of strong appeal. The second aspect is the quantity and quality of input features. In a typical microbiome study, there are often thousands of features from the otus, and the number of features greatly exceeds the number of included samples, making it difficult to accurately include key features, leading to overfitting of the model and reducing the accuracy of the model classification. Although a large number of efficient feature selection methods are used to select the most relevant features as input to the model, it is difficult to overcome this dilemma. However, overfitting can often be prevented by fitting multiple models and using multiple validation sets or cross-validation to compare their predictive accuracy on the test data. Alternatively, overfitting can be reduced by training the model on a larger number of data points. The third challenge is the generalization ability of the ML model, that is, the model developed should apply not only to the training data set, but also to other similar data sets. At present, relatively few studies on the application of ML in cancer microbiomics involve validation of training models with external data and multi-center datasets. Therefore, model validation in multiple external data sets or multi-center data sets will be an effective means to avoid the generation of overfitting models and evaluate the generalization ability of models efficiently. Finally, due to the limitation of detection technology, the microbial information in a large number of solid tumors has not been deeply mined, so high-precision detection technology is still expected.

In addition to the challenges mentioned above, there is a greater requirement to focus on analyzing the quality of the data and the processing of research results with high AUC. In a study by Poore et al. (2020), the authors demonstrated the ability to differentiate between multiple cancer types using only nucleic acids from blood and tissue microbes by using ML algorithms, with most of their models exceeding 95% accuracy. Due to the large size of the study, its methodology was fairly rigorous from the description of the article, while obtaining very satisfactory results, thus making it susceptible to imitation by a large number of scholars. However, the study did make some errors in the analysis of the data. First, there were significant errors in the normalization of the raw data read counts. Poore et al. (2020) used normalization rather than raw data to construct their machine learning classifier to eliminate batch effects. However, during the standardization process, many cancer types were labeled with incorrect values, and these incorrect manual labels were used to create highly accurate classifiers. The Voom-SNM normalization approach used by Poore et al. (2020) would inadvertently append *a priori* information about tumor type to the normalized data. In order to address such false-positive events, it is necessary to ensure the credibility of the results by choosing the appropriate standardized approach for different data types, as well as appropriate pre-experiments to verify their feasibility. Secondly, Poore et al. (2020) incorrectly categorized human reads as bacteria. These human readings matched to bacteria were unrelated to the actual presence of bacteria in the tumor samples, leading to millions of false-positive findings for bacterial readings. To eliminate this interference, reads not mapped to the human genome can be compared to the complete CHM13 human genome using a sequence comparison tool such as Bowtie2 to exclude additional reads with human matches. Beyond that, a great deal of the data and findings in this article need to be confirmed one by one. Similar major data analysis errors need to be reduced in future studies of the microbiome and ML to avoid invalid findings.

In cancer and microbiota-related studies, more attention should be paid to regions other than the gut microbiota, such as the oral cavity, skin, breast, abdominal cavity, urine, and reproductive tract, because studies of these specific microbiota are still scarce. Also, there is a need to think about how to use ML tools to process microbiome data and how to describe the relationship between training data, test data, overfitting and generalization. In the future, researchers should actively embrace the artificial intelligence revolution and become ML practitioners, leveraging research findings in computer science to achieve the use of microbiota analysis as a powerful tool for disease prevention, prediction, diagnosis and treatment.

## Conclusion

Despite the many challenges, ML remains an important tool in the study of cancer and microbiota, and it represents a critical step in the search for sensitive, specific and non-invasive methods of cancer diagnosis. In short, researchers have used machine learning to excavate a large number of microorganisms with potential links to the development of a variety of tumors, which will promote the development of new tumor markers.

## Supplemental Information

10.7717/peerj.16304/supp-1 Supplemental Information 1 Supplementary documents. No original data were available for this literature review, and all data sources were searched in the PubMed and Web of Science online database.

## Abbreviations

ML: Machine learning
NGS: Next generation sequencing
mNGS: Metagenomic NGS
OTU: Operational taxonomic unit
RF: Random forest
LR: Logistic regression
SVM: Support vector machine
DL: Deep learning
ANN: Artificial neural networks
SOM: Self-organizing map
PCA: Principal components analysis
PCoA: Principal coordinate analysis
t-SNE: T-distributed stochastic neighbor embedding
TCGA: The Cancer Genome Atlas
WGS: Whole genome sequencing
TL: Transfer learning
CRC: Colorectal cancer
FOBT: Fecal occult blood testing
CEA: Carcinoembryonic antigen
LEfSe: Linear discriminant analysis of effect sizes
CRA: Colorectal adenoma
IBD: Inflammatory bowel disease
LDA: Linear discriminant analysis
NB: Naïve Bayesian
LASSO: Last absolute shrinkage and selection operator
GBM: Gradient boosting machine; FDR, false discovery rate
GMSM: Gut microbiome-associated serum metabolites
N: Normal healthy population
LC: Lung cancer
mRMR: Minimum-redundancy maximum-relevancy
rRNA: Ribosomal RNA
ROC: Receiver operating characteristic
AUC: Area under the curve

## Additional Information and Declarations

### Data Availability

The following information was supplied regarding data availability: This is a literature review and hence did not utilize raw data.
