# Supplementary material for: Machine learning: a powerful tool for identifying key microbial agents associated with specific cancer types
Source: PeerJ. 2023 Oct 23;11:e16304. doi: 10.7717/peerj.16304 (PMC10601900; doi:10.7717/peerj.16304)
Supplement: Supplemental Information 1 — No original data were available for this literature review, and all data sources were searched in the PubMed and Web of Science online database. [file peerj-11-16304-s001.docx]

No original data were available for this literature review, and all data sources were searched in the pubmed and Web of Science online database.
